# Outcomes of 25-Gauge Vitrectomy for Tractional and Nontractional Diabetic Macular Edema with Proliferative Diabetic Retinopathy

**DOI:** 10.1155/2019/5304524

**Published:** 2019-12-11

**Authors:** Hideaki Someya, Kei Takayama, Masaru Takeuchi, Hiroshi Yokoyama, Takeshi Kimura, Masakazu Morioka, Yoshihiro Takamura, Seiji Sameshima, Tetsuo Ueda, Nahoko Ogata, Maki Tashiro, Shigehiko Kitano, Taiji Sakamoto

**Affiliations:** ^1^Department of Ophthalmology, National Defense Medical College, Tokorozawa 359-8513, Japan; ^2^Department of Ophthalmology, University of Fukui Faculty of Medical Sciences, Yoshida 910-1193, Japan; ^3^Japan Clinical Retina Study Group, Kagoshima, Japan; ^4^Department of Ophthalmology, Hyogo College of Medicine, Nishinomiya 634-8521, Japan; ^5^Department of Ophthalmology, Kagoshima University, Kagoshima 890-8544, Japan; ^6^Department of Ophthalmology, Nara Medical University, Kashihara 634-8522, Japan; ^7^Diabetes Center, Tokyo Women's Medical University School of Medicine, Tokyo 162-8666, Japan

## Abstract

The benefit of pars plana vitrectomy with internal limiting membrane peeling for tractional macular edema and diffuse nontractional macular edema in diabetic retinopathy has been reported. Although these studies had included various stages, use of conventional 20-gauge vitrectomy system, small number of cases, single-center study, and lack of retinal structure measurements were limitations. We compared one-year outcomes of 25-gauge vitrectomy for refractory diabetic macular edema with or without the tractional proliferative membrane in proliferative diabetic retinopathy (PDR) eyes and examined the prognostic factors for postoperative visual acuity. A total of consecutive 116 PDR eyes of 116 patients that underwent 25-gauge vitrectomy for tractional macular edema (TME group: 56 eyes) or nontractional macular edema (nTME group: 60 eyes) at six centers were retrospectively reviewed. Visual acuity (VA), central macular thickness (CMT), complications, and postoperative treatments before and 12 months after vitrectomy were compared. Mean VA improved significantly in each group (both *P* < 0.01), and mean CMT decreased significantly in each group (both *P* < 0.01). Thirteen eyes underwent additional vitrectomy, six eyes developed neovascular glaucoma, six eyes received intravitreal anti-VEGF injection, and thirteen eyes received subtenon triamcinolone acetonide injection. Multiple linear regression analysis showed that baseline VA and CMT in the TME group and kidney function in the nTME group were the predictable factors of the 12-month postoperative VA. Twenty-five-gauge vitrectomy effectively improved VA and macular structure both in TME and nTME groups. Baseline VA, CMT, and kidney function are important factors affecting postoperative VA.

## 1. Introduction

Diabetic macular edema (DME) is a common cause of visual loss in diabetic retinopathy (DR) and is a complication in any stages of DR including proliferative diabetic retinopathy (PDR) [1]. The current gold standard treatment for central-involving DME is intravitreal antivascular endothelial growth factor (anti-VEGF) therapy [2, 3]. However, tractional macular edema (TME) might be treated by vitrectomy with membrane removal since traction is not resolved by intravitreal anti-VEGF therapy [4]. Vitrectomy is also useful in some cases of diffuse nontractional macular edema (nTME) resistant to laser treatment or pharmacotherapy because vitrectomy improves oxygenation of the retina and diffusion of nutrients between the vitreous and retina [5–8].

Compared to the 20-gauge vitrectomy system, microincision transconjunctival sutureless vitrectomy systems decrease surgical invasion, shorten the operating time and duration of hospitalization, and lower the incidence of intra- and postoperative complications [9–11]. Vitrectomy combined with phacoemulsification and implantation of the intraocular lens is also effective for removal of peripheral vitreous gel and has advantages in some patients with PDR [12]. Furthermore, postoperative outcomes are further improved by preoperative adjunctive intravitreal anti-VEGF injection [13], internal limiting membrane peeling (ILMP) [14, 15], vitreous visualization using triamcinolone acetonide [16], and wide-angle viewing system [17].

Although previous studies have shown the benefit of vitrectomy for TME or nTME in diabetic retinopathy [18, 19], these studies had several limitations such as inclusion of various stages of diabetic retinopathy, use of the conventional 20-gauge vitrectomy system, small number of cases, retrospective design, and single-center study. In the present study, we compared real-world outcomes of visual acuity and anatomical restoration in the eyes with TME and nTME that underwent vitrectomy using current techniques and procedures and identified preoperative prognostic factors affecting postoperative visual acuity.

## 2. Materials and Methods

This study was an observational case series of 116 eyes of 116 consecutive participants with type I (4 participants) or II (112 participants) diabetic mellitus who underwent primary 25-gauge vitrectomy for PDR with DME in six centers (National Defense Medical College Hospital; Hyogo College of Medicine Hospital; University of Fukui Hospital; Kagoshima University Hospital; Nara Medical University Hospital; and Tokyo Women's Medical University School of Medicine Diabetes Center) between April 2010 and March 2016. The study was conducted according to the tenets of the Declaration of Helsinki and was approved by institutional review board of each of the six hospitals. Inclusion criteria were as follows: (1) All participants had intravenous fluorescein angiograms before vitrectomy or after vitrectomy to confirm the diagnosis of PDR by two ophthalmologists. In cases with vitreous hemorrhage (VH) which obscures complete fundus observation before vitrectomy, two ophthalmologists confirmed the diagnosis of PDR by fundus observation and fluorescein angiograms after vitrectomy. (2) All participants were candidates for vitrectomy and signed a preoperative informed written consent form for the surgical procedure. (3) Participants with follow-up periods more than 12 months. Exclusion criteria were as follows: (1) participants with a follow-up period of less than 12 months, (2) other causes of proliferative vitreoretinal disease, (3) central retinal ischemia detected by intravenous fluorescein angiograms, (4) having a past history of vitrectomy, (5) having a past history of other retinal disease such as retinal vein occlusion and age-related macular degeneration, and (6) having a past history of any type of glaucoma.

All eyes were classified into TME group and nTME group. TME contained the proliferative membrane around the macula which attached the fovea and macular edema, while nTME lacked the proliferative membrane attaching to the fovea and macular edema. This classification was decided by at least 2 retinal specialists in each center at the primary vitrectomy based on preoperative fundus examination, color photographs, spectral domain-optical coherence tomography (SD-OCT) findings, and ultrasonography or intraoperative findings as described previously [20, 21].

### 2.1. Surgical Procedures and Postoperative Treatment

All eyes underwent 25-gauge vitrectomy using a wide-angle viewing system. In brief, the posterior vitreous was separated from the retina by active aspiration with the vitrectomy probe, and all visible vitreous strands that adhered to the retina were removed. Intravitreal triamcinolone acetonide (40 mg/mL, MaQaid; Wakamoto pharmaceutical Co., Tokyo, Japan) was systematically used in all cases to facilitate visualization and removal of the adherent posterior cortical vitreous. ILMP was systematically performed by staining the ILM with brilliant blue *G* followed by removal. Eyes with insufficient previous panretinal photocoagulation received additional intraoperative panretinal photocoagulation. No eye had received intravitreal anti-VEGF injection or intravitreal/subtenon triamcinolone acetonide injection at the end of vitrectomy. After vitrectomy, topical antibiotic and anti-inflammatory agents were administered 4 times daily for 1 month, and postoperative additional treatments, i.e., revitrectomy, intravitreal anti-VEGF injection, subtenon triamcinolone acetonide (STTA) injection, and cataract surgery, were performed by the clinician's decision, but no eye had received intravitreal triamcinolone acetonide injection.

### 2.2. Measurement of Visual Acuity, Intraocular Pressure, and Retinal Structure

At each visit, the patients underwent a complete ophthalmologic examination including best-corrected visual acuity, refractive measurement, SD-OCT, intraocular pressure measurement, slit-lamp examination, and dilated fundus examination (using contact and noncontact fundus lenses). Visual acuity was measured using the standard Japanese decimal visual acuity chart, and the values were converted to logarithm of the minimum angle of resolution (LogMAR) units for data analysis, as described previously [22]. Intraocular pressure was measured by noncontact tonometry or Goldmann applanation tonometer, and ocular hypertension was defined as intraocular pressure >21 mmHg. SD-OCT scans were performed preoperatively to measure the retinal thickness and to evaluate the presence of macular edema and/or macular traction. Central macular thickness (CMT) measurements were read from the automated measurements generated by the machine using the retinal map analysis protocol.

### 2.3. Comparison of Ocular Factors and General Factors

Baseline general conditions (age, sex, diabetic duration, HbA1c, estimated glomerular filtration rate (eGFR), systemic hypertension, and anticoagulant therapy), baseline ocular conditions (LogMAR, CMT, phakia or pseudophakia, ocular hypertension, vitreous hemorrhage, preoperative adjunctive intravitreal anti-VEGF injection, and retinal photocoagulation), surgical procedures (combined phacoemulsification, retinal break, and tamponade material), postoperative complications (vitreous hemorrhage, neovascular glaucoma, and cataract formation), and postoperative additional treatments (revitrectomy, intravitreal anti-VEGF injection, and STTA) were evaluated. HbA1c is expressed in the National Glycohemoglobin Standardization Program (NGSP) unit [23]. Hypertension was defined as use of the antihypertension agent or diagnosed by physicians in each hospital.

### 2.4. Statistical Analysis

Results are expressed as mean ± standard deviation with range [low–high] for continuous variables. The Mann–Whitey *U* test and chi-square test were used to compare the data between the two groups, and repeated measures ANOVA and the Bonferroni correction were used to compare the changes in LogMAR and CMT in the group. Multiple linear regression analysis was used to detect the correlation between the change in LogMAR and other factors. A *P* value less than 0.05 was considered significant.

## 3. Results

### 3.1. Participants and Treatment

Baseline characteristics, surgical procedures, and postoperative complications and additional treatments in TME and nTME groups are shown in Table 1.

The TME group consisted of 56 eyes of 56 patients (36 males; mean age 57.8 ± 9.5 years [38–74]), and the nTME group comprised 60 eyes of 60 patients (36 males; mean age 58.7 ± 14.1 years [30–81]). There were significant differences in vitreous hemorrhage (*P* < 0.001) but no significant differences in baseline general conditions between two groups. In surgical procedures, combined cataract surgery was performed more frequently in the TME group than that in the nTME group (*P* < 0.001). However, the eyes with intraocular lens (IOL) were 50 eyes in the TME group and 47 eyes in the nTME group at 12 months after vitrectomy, and statistical difference was not observed between the two groups. In postoperative complications, 13 eyes (8 eyes in the TME group and 5 eyes in the nTME group) underwent revitrectomy, and 5 eyes (1 eye in the TME group and 4 eyes in the nTME group) developed postoperative NVG. In additional treatment, 2 eyes (3 months and 10 months after vitrectomy) received intravitreal anti-VEGF injection and 10 eyes (7 eyes at 1 month, 2 eyes at 4 months, and 1 eye at 6 months after vitrectomy) received STTA injection in the TME group, and 4 eyes (1 eye at 1 month, 1 eye at 3 months, and 2 eyes at 6 months after vitrectomy) received intravitreal anti-VEGF injection and 3 eyes at 1 month after vitrectomy received STTA injections in the nTME group.

### 3.2. Visual and Anatomical Results

**Figure 1:**
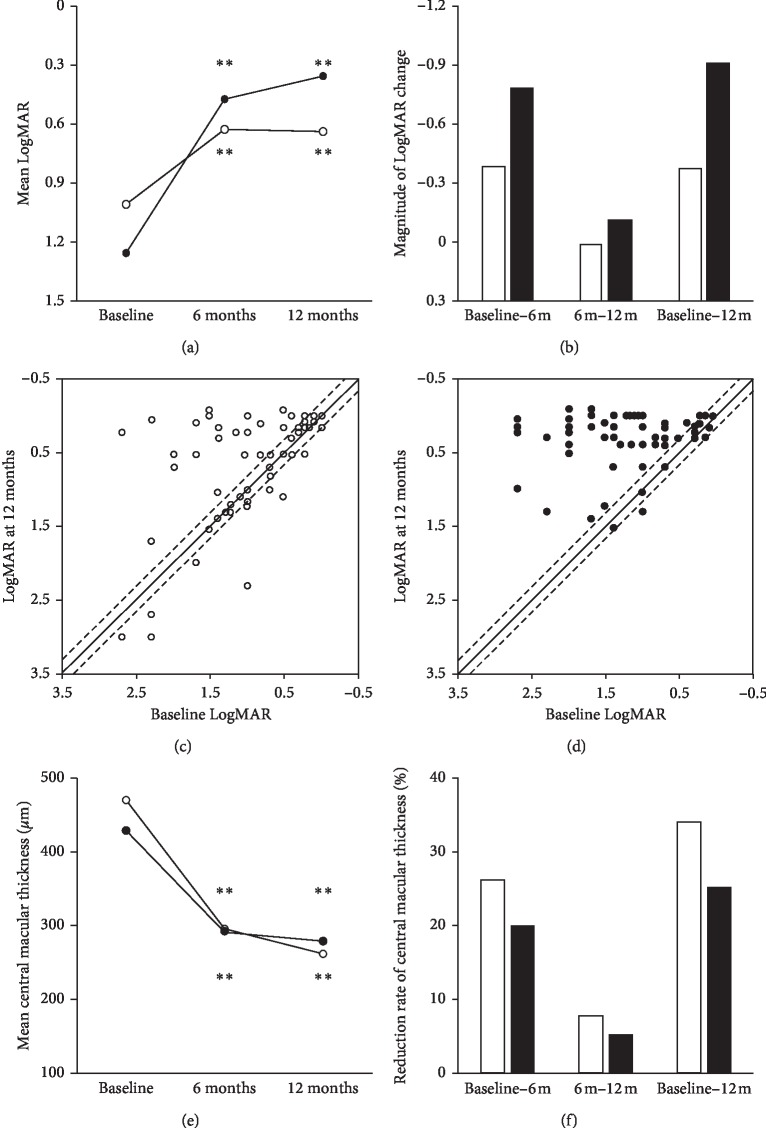
Changes of visual acuity and central macular thickness in two groups. Changes of mean LogMAR (a); magnitudes of LogMAR change (b); plot of baseline LogMAR and 12-month postoperative LogMAR in TME and nTME groups (c, d); changes of mean central macular thickness and magnitudes of central macular thickness change in TME (e) and nTME groups (f). Circle and white bar mean the TME group, and black dots and black bar mean the nTME group. Dash lines mean the changes of 0.2 LogMAR (corresponding to 2 lines). ^*∗∗*^*P* < 0.001.

Mean LogMAR in the TME group and nTME group improved significantly from 1.05 ± 0.71 [2.7–0] and 1.26 ± 0.74 [2.7–0.05], respectively, at baseline to 0.66 ± 0.65 [2.7 to −0.08] and 0.48 ± 0.55 [2.3 to −0.08] (both *P* < 0.001) at 6 months, and 0.69 ± 0.78 [3.0 to −0.08] and 0.36 ± 0.41 [1.5 to −0.08] (both *P* < 0.001) at 12 months (Figure 1(a)).

The mean visual improvement in the TME group and nTME group was 0.39 LogMAR (corresponding to a mean increase of 3.9 lines) and 0.78 LogMAR (mean increase of 7.8 lines), respectively, from baseline to 6 months, −0.03 LogMAR (mean decrease of 0 lines) and 0.12 LogMAR (mean increase of 1.2 lines) from 6 months to 12 months, and 0.36 LogMAR (mean decrease of 3.6 lines) and 0.90 LogMAR (mean increase of 9.0 lines) from baseline to 12 months, respectively (Figure 1(b)). In the TME group, LogMAR improved by more than 2 lines in 23 (41.1%) eyes, did not change by more than 2 lines in 24 eyes (42.9%), and worsened by more than 2 lines in 9 eyes (16.1%) (Figure 1(c)). In the nTME group, LogMAR improved by more than 2 lines in 47 (78.3%) eyes, did not change by more than 2 lines in 12 eyes (20.0%), and worsened by more than 2 lines in 1 eye (1.7%) (Figure 1(d)). Comparing 2-line LogMAR improvement, there was significant difference between two groups (*P* < 0.001).

However, when LogMAR improvement from baseline to 12 months was compared in two groups, there was no significant difference (*P*=0.058). Subsequently, we evaluated the differences between eyes with and without visual improvement in TME and nTME groups (Table 2).

In the TME group, there were significant differences in baseline LogMAR (*P*=0.003), CMT (*P*=0.005), and VH (*P*=0.019) between improved and nonimproved eyes. In the nTME group, there were significant differences in baseline LogMAR (*P*=0.001) and VH (*P*=0.037). We then evaluated the differences between LogMAR at 12 months better and worse than 1.0 (Table 3).

In the TME group, eyes with LogMAR better than 1.0 (*n* = 40) had better baseline LogMAR (*P*=0.002) than eyes with LogMAR worse than 1.0 (*n* = 16). In the nTME group, eyes with LogMAR better than 1.0 (*n* = 51) were younger (*P*=0.0017), had shorter diabetic duration (*P* = 0.0069), and had better baseline LogMAR (*P*=0.039) than eyes with LogMAR worse than 1.0 (*n* = 9).

Mean CMT in the TME group and nTME group decreased significantly from 467 ± 200 [150–1008] and 410 ± 133 [148–998] *μ*m, respectively, at baseline to 291 ± 129 [109–707] and 292 ± 99 *μ*m [135–610] (both *P* < 0.01) at 6 months and 259 ± 109 [195–610] and 277 ± 110 *μ*m [101–700] (both *P* < 0.01) at 12 months (Figure 1(e)). The mean percent CMT reduction in the TME group and nTME group was 29.0% and 22.9%, respectively, from baseline to 6 months, 7.5% and 2.9% from 6 months to 12 months, and 29.0% and 22.9% from baseline to 12 months (Figure 1(f)). There was a no significant difference in postoperative improvement of CMT between the two groups.

### 3.3. Prognostic Factors for 12-Month Visual Acuity

We conducted multiple linear regression analyses to examine the relationship between LogMAR at 12 months and baseline LogMAR or VH in the TME group and baseline LogMAR, diabetic duration, VH, and eGFR in the nTME group, which might affect postoperative LogMAR in the TME group and nTME group (Tables 2 and 3) and age and sex from general condition. A positive correlation with baseline LogMAR (*P*=0.001, 95% confidence interval (CI): 0.24–0.75) and baseline CMT (*P*=0.031, 95% CI: 0.000–0.002) and negative correlation between VH (*P*=0.039, 95% CI: −0.75–0.02) were observed in the TME group, while a positive correlation with diabetic duration (*P*=0.017, 95% CI: 0.002–0.023) and a negative correlation with eGFR (*P*=0.003, 95% CI: −0.007–0.002) were observed in the nTME group (Table 4).

## 4. Discussion

The present study found no significant differences in the improvement of LogMAR and reduction of CMT after microincision transconjunctival sutureless vitrectomy with ILMP between TME and nTME in the eyes with PDR. Approximately 40% of TME patients and 80% of nTME patients had visual gain of more than 2 lines at 12 months. Furthermore, no significant differences in surgical complications were found between TME and nTME. Finally, baseline LogMAR and CMT in TME and baseline LogMAR and kidney function in nTME were identified as independent predictive factors of the final LogMAR.

The effectiveness of vitrectomy for tractional DME was demonstrated by Lewis et al. [24] in 1992 and was confirmed by further prospective studies. The http://DRCR.net prospective study on vitrectomy outcomes in the eyes with diabetic macular edema and vitreomacular traction reported significant visual improvement in 38% and significant visual deterioration in 22% of the subjects [4]. In the present study, vitrectomy with ILMP was performed for TME or nTME involved in eyes in which PDR was evaluated, and the DME eyes with non-PDR eyes were excluded. Visual improvement and deterioration in tractional DME were 23 eyes (41.1%) versus 9 eyes (16.1%), which was similar to the results of the http://DRCR.net prospective study [4]. However, LogMAR improvement was achieved by resolving both DME and various preoperative complications in this study. For instance, preoperative VH, which could account for visual disturbance, was observed in 52 eyes, and combined cataract surgery was performed in 36 eyes out of 60 eyes with nTME. Although multiple linear regression analysis showed no significant correlation between 12-month LogMAR and preoperative VH and combined cataract surgery, these could be considered as the causes that mean visual improvement at 12-month follow-up in both groups was higher than previous studies of vitrectomy with ILMP on DME [4, 24].

In clinical situation, there is a good deal of evidence suggesting that vitrectomy effectively restores retinal function and significantly decreases macular edema. Lewis et al. reported that vitrectomy was effective in the eyes with macular edema associated with a thickened and taut posterior hyaloid membrane [24]. Although the role of vitrectomy in the treatment of DME remains not to be fully elucidated, several physiopathologic mechanisms have been suggested to explain the favorable results obtained with vitrectomy in the nTME eyes. Vitrectomy removes vitreous cortex adjacent to the retina, improves retinal oxygenation, eliminates inflammatory cells, and reduces inflammatory cytokines and proangiogenic factors such as VEGF that induces DME [25, 26]. In this study, all eyes underwent ILMP. The rationale for this procedure is that ILMP allows complete removal of overlying residual vitreous cortex and inflammatory cells adhering to the inner surface of the ILM [27, 28].

Vitrectomy for PDR is associated with certain complications, including postoperative VH, cataracts progression, IRT, retinal detachment, and NVG [9, 13, 19]. Postoperative VH is a most frequent complication, and Wakabayashi reported the percentage of postoperative VH in 60 PDR eyes during 6-month follow-up was 25% [29], and Ozone reported that percentage of postoperative VH was 22% [30]. NVG is the most severe complication following vitrectomy. Kumagai and Yamamoto reported the incidence of developing postoperative NVG were 3.9% and 4.6% [5, 31]. In the present study, postoperative VH was 27 eyes (23%) and NVG was 6 eyes (5.1%) that were compatible with those of previous reports. On the contrary, it was reported that more than one-half of phakic eyes developed cataract in conventional vitrectomy for DME [18]. However, visual disturbance by cataract progression is avoidable. In Japan, cataract surgery is often combined with vitrectomy to prevent the visual loss that occurs with progression of cataract [32]. As well as in this study, 85 eyes (73.3%) underwent combined cataract surgery. Except for 13 eyes (11.2%) which had undergone past cataract surgery, 18 eyes (15.6%) remained phakic; however, 5 of 18 phakia eyes (27.8%) needed additional cataract surgery within 6 months. This result might suggest that combined cataract surgery is preferable to prevent high proportion of postoperative cataract progression.

Renal dysfunction is associated with an increased likelihood of worsening diabetic retinopathy [33]. A retrospective study from the US which involves more than 4000 patients with diabetic retinopathy showed the presence of nephropathy increased the risk of progression to proliferative diabetic retinopathy [34]. Diabetic kidney dysfunction decreases the secretion of erythropoietin that causes chronic anemia and retinal tissue and cellular hypoxia [35], and kidney dysfunction is known to be a risk factor for developing diabetic retinopathy [36] and DME [37]. Management of diabetic retinopathy in patients at risk of severe acute renal impairment should follow more strict and aggressive rules [38]. In the present study, multiple linear regression analysis identified kidney function as a factor negatively correlated with postoperative visual function in the nTME group, consistent with previous report [37]. This multicenter study also demonstrates kidney function as an important factor for diabetic retinopathy.

In the surgical procedure, all eyes had received intravitreal triamcinolone injection and many eyes had additional retinal photocoagulation. Intravitreal triamcinolone and retinal photocoagulation had some effects for the postoperative retinal structure and even visual outcomes in short term and would not continue after 6 months. Additional anti-VEGF and STTA injections were performed in 19 eyes. In the two groups, anti-VEGFs were performed at 1 month, 3 months, 6 months, and 10 months after vitrectomy. STTA injections were performed at 1 month, 3 months, and 6 months. In general, these treatments present immediate effects and do not continue for a long time. However, it is possible that the efficacy might affect the visual and anatomical outcomes at 6 months or 12 months in some cases.

This study has several limitations. The major limitation is the retrospective multicenter comparative study without any control group although all surgical procedures were performed under similar conditions. Baseline factors which affect postoperative LogMAR, such as lens status, anti-VEGF and STTA injections, and VH, were evaluated although macular degeneration or optic nerve atrophy could not be standardized or excluded. A second limitation is that only Japanese subjects were studied and both type 1 and type 2 diabetes mellitus were included. Finally, the absence of difference between the two groups may be simply attributable to the lack of statistical power in this study or to selection bias.

## 5. Conclusion

This study confirmed the functional and anatomical efficacy of vitrectomy for both tractional and nontractional DME in PDR eyes, without severe surgical complications. The real-world outcomes obtained from this multicenter study are more reliable than previous single-center studies. The high and increasing prevalence of DME and the existence of nonresponders to anti-VEGF therapy require consideration of alternative therapies such as vitrectomy that may improve diabetic patient care.

## Figures and Tables

**Table 1:** Baseline characteristics, surgical procedures, and postoperative complications and additional treatments in the two groups.

	TME	nTME	*P*
Baseline general conditions			
Eyes	56	60	
Mean age (year)	57.8 ± 9.5 [38–74]	58.7 ± 14.1 [30–81]	0.20
Sex (male/female)	36/20	36/24	0.63
Diabetic duration (years)	12.4 ± 11.7 [1–41]	10.6 ± 9.2 [1–46]	0.38
HbA1c (%)	7.6 ± 1.7 [5.3–13.3]	7.9 ± 1.5 [4.8–11.8]	0.26
eGFR (ml/m)	54.9 ± 33.0 [0.5–112]	58.8 ± 36.8 [0.5–111]	0.25
Hypertension	34	43	0.21
Anticoagulant use	7	4	0.45

Baseline ocular conditions			
Mean LogMAR	1.05 ± 0.71 [2.7–0]	1.26 ± 0.74 [2.7–0.05]	0.06
Mean CMT (*μ*m)	467 ± 200 [150–1008]	410 ± 133 [148–998]	0.06
Lens status (IOL/phakia)	3/53	10/50	0.10
Ocular hypertension	4	1	0.32
Vitreous hemorrhage	31	52	<0.001
Tractional retinal detachment	11	5	0.13
Adjunctive intravitreal anti-VEGF injection	16	11	0.19
Retinal photocoagulation	39	46	0.39

Surgical procedures			
Combined cataract surgery	49	36	<0.001
Retinal break	17	9	0.079
Tamponade (air/gas/silicon oil)	18/8/1	16/6/0	0.54

Postoperative complications and additional treatments			
Vitreous hemorrhage	13	14	0.99
Neovascular glaucoma	2	4	0.40
Cataract progression	1	4	0.21
Revitrectomy	8	5	0.47
Intravitreal anti-VEGF injection	2	4	0.74
Subtenon steroid injection	10	3	0.057

Data are expressed as number of eyes or patients or mean ± standard deviation [low–high].

**Table 2:** Comparison of the eyes with and without visual acuity improvement in the two groups.

	Improvement	No improvement	*P*
TME group	23 eyes	33 eyes	
Baseline general conditions			
Mean age (year)	55.5 ± 9.4 [38–69]	59.0 ± 9.2 [40–74]	0.09
Sex (male/female)	12/11	24/9	0.19
Diabetic duration (year)	11.0 ± 11.0 [1–30]	12.5 ± 12.3 [1–41]	0.27
HbA1c (%)	7.4 ± 1.9 [5.3–11.6]	7.9 ± 1.7 [6.4–13.3]	0.059
eGFR (ml/m)	55.0 ± 28.7 [0.93–96]	56.7 ± 35.8 [0.5–112]	0.48
Hypertension	16	18	0.26
Anticoagulant use	5	2	0.18
Baseline ocular conditions			
Mean LogMAR	1.34 ± 0.65 [2.7–0.2]	0.83 ± 0.70 [2.7–0]	0.003
Mean CMT (*μ*m)	603 ± 162 [356–801]	413 ± 195 [150–1008]	0.005
Lens status (IOL/phakia)	0/23	3/30	0.25
Ocular hypertension	1	3	0.38
Vitreous hemorrhage	17	14	0.019
Tractional retinal detachment	6	5	0.31
Adjunctive intravitreal anti-VEGF injection	8	8	0.58
Retinal photocoagulation	14	25	0.23

nTME group	47 eyes	13 eyes	
Baseline general conditions			
Mean age (year)	57.9 ± 13.9 [30–77]	61.8 ± 15.0 [42–81]	0.18
Sex (male/female)	27/20	9/4	0.65
Diabetic duration (years)	9.5 ± 8.3 [1–30]	14.7 ± 14.1 [1–46]	0.18
HbA1c (%)	8.0 ± 1.8 [4.8–11.8]	7.5 ± 1.0 [6.4–10.2]	0.19
eGFR (ml/m)	61.6 ± 41.0 [0.5–111]	66.1 ± 40.8 [0.55–110]	0.32
Hypertension	33	10	0.90
Anticoagulant use	3	1	0.64
Baseline ocular conditions			
Mean LogMAR	1.47 ± 0.67 [2.7–0.22]	0.51 ± 0.45 [1.4–0.05]	<0.001
Mean CMT (*μ*m)	429 ± 186 [148–811]	363 ± 97 [242–988]	0.19
Lens status (IOL/phakia)	12/35	2/11	0.69
Ocular hypertension	1	0	0.21
Vitreous hemorrhage	43	9	0.037
Tractional retinal detachment	4	1	0.92
Adjunctive intravitreal anti-VEGF injection	10	1	0.06
Retinal photocoagulation	36	10	0.98

Data are expressed as number of eyes or patients or mean ± standard deviation [low–high].

**Table 3:** Comparison of eyes with 12 months LogMAR better versus worse than 20/200 in the two groups.

	Better	Worse	*P*
TME group	40 eyes	16 eyes	
Baseline general conditions			
Mean age (year)	57.8 ± 9.3 [38–74]	57.8 ± 10.1 [40–70]	0.13
Sex (male/female)	24/16	12/4	0.45
Diabetic duration (years)	12.0 ± 11.7 [1–41]	13.5 ± 13.7 [1–40]	0.29
HbA1c (% (mmol/mol))	7.8 ± 2.1 [5.3–13.3]	7.4 ± 0.9 [5.6–9.3]	0.13
eGFR (ml/m)	55.4 ± 34.8 [0.61–112]	53.8 ± 30.0 [0.5–101]	0.47
Hypertension	27	7	0.18
Anticoagulant use	4	3	0.65
Baseline ocular conditions			
Mean LogMAR	0.87 ± 0.68 [2.7–0]	1.50 ± 0.60 [2.7–0.52]	0.002
Mean CMT (*μ*m)	475 ± 154 [227–801]	451 ± 290 [150–1008]	0.16
Lens status (IOL/phakia)	1/39	2/14	0.19
Ocular hypertension	2	2	0.57
Vitreous hemorrhage	24	7	0.41
Tractional retinal detachment	7	4	0.52
Adjunctive intravitreal anti-VEGF injection	11	5	0.96
Retinal photocoagulation	27	12	0.58

nTME group	51 eyes	9 eyes	
Baseline general conditions			
Mean age (year)	56.4 ± 13.8 [30–77]	71.8 ± 7.5 [58–81]	0.0017
Sex (male/female)	30/21	6/3	0.66
Diabetic duration (years)	9.0 ± 8.3 [1–30]	23.0 ± 14.3 [7–46]	0.0069
HbA1c (% (mmol/mol))	8.0 ± 1.6 [4.8–11.8]	7.3 ± 1.4 [6.0–10.2]	0.13
eGFR (ml/m)	66.2 ± 41.0 [0.5–111]	40.7 ± 31.7 [0.6–71]	0.06
Hypertension	36	6	0.87
Anticoagulant medication	4	1	0.74
Baseline ocular conditions			
Mean LogMAR	1.18 ± 0.72 [2.7–0.05]	1.70 ± 0.70 [2.7–1]	0.039
Mean CMT (*μ*m)	408 ± 176 [145–811]	408 ± 105 [247–501]	0.42
Lens status (IOL/phakia)	8/43	2/7	0.63
Ocular hypertension	0	1	0.15
Vitreous hemorrhage	42	6	0.53
Tractional retinal detachment	3	2	0.10
Adjunctive intravitreal anti-VEGF injection	10	1	0.89
Retinal photocoagulation	40	6	0.74

Data are expressed as number of eyes or patients or mean ± standard deviation [low–high].

**Table 4:** Prognostic factors affecting visual acuity at 12 months after vitrectomy in the two groups.

	Variable	Estimate	95% CI	*P*
TME group				
Adjusted	Age	−0.005	−0.02–0.01	0.58
*R*^2^ = 0.44	Sex	−0.32	−0.68–0.03	0.07
*P* < 0.001	Baseline LogMAR	0.50	0.24–0.75	<0.001
	Vitreous hemorrhage	−0.38	−0.75–0.02	0.039
	Baseline CMT	0.0012	0.000–0.002	0.031

nTME group				
Adjusted	Age	0.001	−0.006–0.008	0.78
*R*^2^ = 0.42	Sex	−0.029	−0.22–0.16	0.76
*P* < 0.001	Baseline LogMAR	0.097	−0.028–0.22	0.13
	Diabetic duration	0.013	0.002–0.023	0.017
	Vitreous hemorrhage	−0.25	−0.54–0.05	0.10
	eGFR	−0.004	−0.007–0.002	0.003

## Data Availability

The data used to support the findings of this study are available from the corresponding author upon request.
